# Engineering the Future of Precision Medicine: A Comprehensive Guide to RNA Therapeutics

**DOI:** 10.3390/cimb48080809

**Published:** 2026-08-11

**Authors:** Konstantina Athanasopoulou, Glykeria N. Daneva, Vasiliki-Ioanna Michalopoulou, Maria R. Stamelou, Panagiotis Tsiakanikas, Panagiotis G. Adamopoulos

**Affiliations:** Department of Biochemistry and Molecular Biology, Faculty of Biology, National and Kapodistrian University of Athens, 15701 Athens, Greece; konnath@biol.uoa.gr (K.A.); gldaneva@biol.uoa.gr (G.N.D.); vimich@biol.uoa.gr (V.-I.M.); maria_stamelou@hotmail.com (M.R.S.); ptsiak@biol.uoa.gr (P.T.)

**Keywords:** mRNA vaccines, antisense oligonucleotides (ASOs), small interfering RNAs (siRNAs), CRISPR/Cas, circular RNAs (circRNAs), aptamers, RNA delivery platforms, lipid nanoparticles (LNPs), targeted therapy

## Abstract

RNA therapeutics have evolved from passive genetic intermediaries into highly programmable platforms, fundamentally transforming the landscape of precision medicine. This comprehensive review examines the molecular architecture and mechanisms of established platforms in the clinical setting, including mRNA, antisense oligonucleotides (ASOs), small interfering RNAs (siRNAs) and aptamers, alongside next-generation platforms, such as CRISPR-guided systems and circular RNAs (circRNAs). Moreover, we discuss strategies to overcome systemic delivery bottlenecks and evaluate advanced non-viral systems, emphasizing lipid nanoparticles (LNPs), polymers and tissue-specific ligand conjugates that facilitate precise intracellular targeting. Furthermore, we explore the clinical expansion of these platforms across infectious diseases, rare genetic disorders, oncology and cardiovascular conditions. Finally, we highlight how the integration of artificial intelligence (AI) and machine learning (ML) redefines the limits of individualized, programmable RNA therapies by accelerating sequence optimization and nanoparticle formulation.

## 1. Introduction

Modern molecular biology has fundamentally shifted our understanding of RNA, revolutionizing early models that viewed it merely as a passive intermediary carrying genetic instructions from DNA to the ribosomes. Today, RNA is recognized for its far more dynamic and multidimensional nature. Its capabilities extend far beyond simple genetic blueprint transfer to include structural scaffolding, catalytic activity and the precise regulation of gene expression. Capitalizing on this expanded RNA functionality, modern biotechnology prioritizes RNA as a key platform for the development of highly tailored therapeutics [1,2]. These RNA-based therapies intervene directly at the RNA level, bypassing the constraints of traditional small-molecule drugs and therapeutic peptides [3].

**Figure 1:**
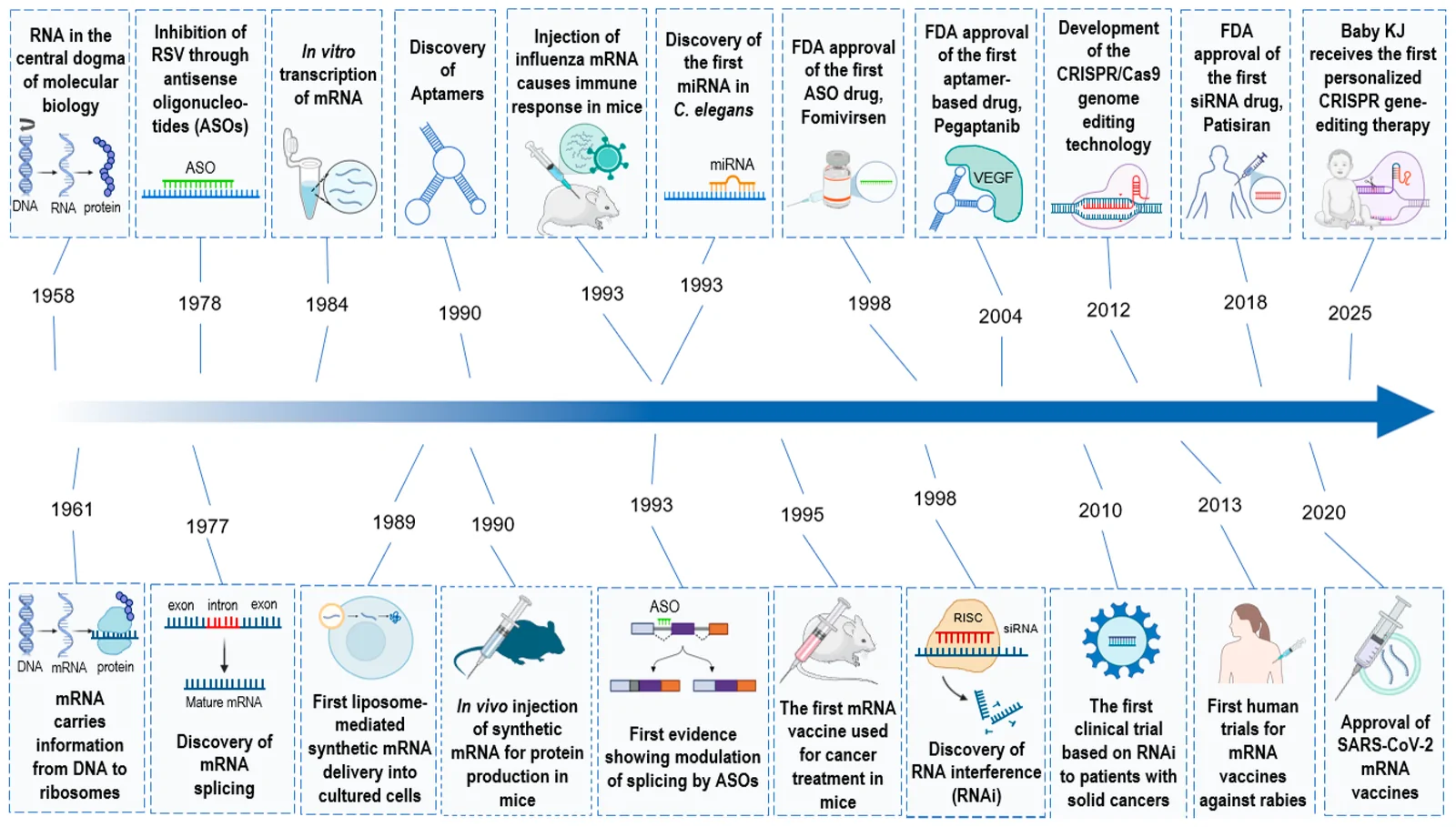
Historical timeline of important events in RNA research and therapeutics.

The transition of RNA from being considered an unstable biological intermediate into a robust therapeutic platform spans over half a century of scientific breakthroughs (Figure 1). Following the discovery of mRNA in 1961, nucleic acids were first used as therapeutic agents in 1978, when Zamecnik and Stephenson successfully used a synthetic antisense oligonucleotide (ASO) to inhibit the Rous sarcoma virus [4]. Two decades later, in 1998, the Food and Drug Administration (FDA) approved the first antisense drug, named Fomivirsen, for the treatment of cytomegalovirus retinitis, validating the clinical utility of engineered nucleic acids [5]. By 1999, Fomivirsen had received European approval and become a critical lifeline for preventing blindness in patients with advanced acquired immunodeficiency syndrome (AIDS), who were highly susceptible to this opportunistic viral infection [6]. In that same year, the discovery of RNA interference (RNAi) unveiled an endogenous cellular mechanism for post-transcriptional gene silencing [7]. While RNAi provided both theoretical and mechanistic frameworks to silence targets that were inaccessible to conventional pharmacotherapy in vivo, translating small interfering RNAs (siRNAs) into clinical reality was initially hindered by biological barriers, such as rapid enzymatic degradation, renal clearance and innate immune activation. To overcome these obstacles, RNA delivery methods were improved, and the RNA structure was altered to include chemical modifications, such as pseudouridine (Ψ) and 2′-O-methylation (Nm), which successfully shielded the RNA from nuclease cleavage and host immune surveillance [8]. The 2018 FDA approval of Patisiran, the first siRNA-based therapy for transthyretin amyloidosis, marked a watershed moment in the clinical translation of RNAi [9,10].

Concurrently, efforts to develop mRNA-based therapies have intensified. Although an early 1990 study demonstrated that injected in vitro-transcribed (IVT) mRNA could express functional proteins in murine muscle, subsequent clinical translation was stalled for decades by the molecule’s extreme instability and severe immunogenicity [11]. Increased immunogenicity can be alleviated by the incorporation of modified nucleosides, particularly Ψ, which reduces Toll-like receptor (TLR) recognition during innate immune responses, while simultaneously increasing stability to enhance translation [12]. To reach their cellular targets, these modified transcripts were encapsulated with advanced ionizable lipid nanoparticles (LNPs) designed to facilitate endosomal escape. This powerful synergy of immunological stealth, high stability, and efficient delivery set the stage for the most rapid pharmaceutical deployment in history: the authorization of the coronavirus disease 2019 (COVID-19) mRNA vaccines in 2020 [13].

Currently, the landscape of RNA therapeutics is expanding beyond classical ASOs and mRNA vaccines, establishing a new paradigm of precision, individualized medicine in oncology and neurodegenerative diseases [14,15]. Next-generation RNA scaffolds including circular RNAs (circRNAs) and self-amplifying RNAs (saRNAs) are overcoming the transient nature of linear mRNA, offering exceptional nuclease resistance and prolonged protein expression [16,17,18]. The field is further expanding through the integration of high-precision, RNA-guided editing technologies, specifically CRISPR/Cas13 systems, to achieve site-specific transcriptome engineering. Unlike traditional DNA-editing platforms, these RNA-targeting ribonucleases enable reversible correction of pathogenic transcripts without the risks of permanent double-strand DNA breaks or long-term genomic toxicity [19]. This approach facilitates a tunable therapeutic window, allowing for the modulation of gene expression with a significantly improved safety profile compared to genomic alterations [20,21].

The integration of advanced nanomaterials, high-throughput sequencing and artificial intelligence (AI) propels the field of RNA therapeutics from a trial-and-error approach into a precision-driven paradigm [22]. The present manuscript explores the current applications and future directions of RNA therapeutics, describing the molecular mechanisms of established platforms, including mRNA and ASOs, alongside next-generation platforms such as circRNAs and CRISPR-guided systems. Furthermore, we examine the strategies employed to overcome systemic delivery bottlenecks, comparing LNPs with next-generation ligand conjugates and viral-free vectors. Finally, we explore the future of precision medicine, where AI-driven methodologies and advanced delivery strategies are set to redefine the limits of programmable therapy, addressing the safety and regulatory frameworks and evaluating potential clinical impact.

## 2. Major Classes and Mechanisms of RNA Therapeutics

### 2.1. mRNA-Based Therapeutics: From Molecular Architecture to Clinical Application

The therapeutic potential of mRNA remained elusive until 1990, when Wolff et al. provided the first in vivo proof of concept that mRNA could be used as a therapeutic tool. When injecting IVT mRNA directly into skeletal muscles of mice, the protein encoded by the mRNA was expressed by the organism’s cells [11]. This discovery paved the way for the development of mRNA vaccines, which was first attempted in 1993. Martinon et al. synthesized in vitro mRNA molecules encoding the influenza virus nucleoprotein and demonstrated that vaccination with liposome-encapsulated mRNA can induce strong virus-specific cytotoxic T lymphocyte (CTL) responses in mice [23]. Despite this breakthrough, the scientific community remained hesitant due to the significant instability and immunogenicity of the delivered mRNA, which triggered severe inflammatory responses. Thorough investigation of the mechanism underlying the immune response mediated by the delivery of exogenous mRNAs demonstrated that uridine replacement with modified nucleosides, such as Ψ, could prevent the recognition of mRNA by TLRs and mitigate the subsequent immune response [24,25]. This principle was successfully transferred into clinical settings with N1-methyl-pseudouridine (m1Ψ) in the highly successful COVID-19 mRNA vaccines [26].

To maximize the therapeutic efficiency of mRNA, the transcripts are meticulously synthesized chemically, by enzymatic transcription, or IVT [27], and exhibit structural resemblance to the endogenous molecules. The IVT mRNA should exhibit marked stability, optimal translation rates and minimal immunogenicity, while the nucleotide sequence and folding should be taken into consideration [28]. The five primary structural elements of an IVT mRNA include the 5′ cap, the 5′ untranslated region (UTR), the coding sequence (CDS), the 3′ UTR, and the poly(A) tail. The CDS engulfs the open reading frame (ORF) and plays a crucial role, as it encodes the desired antigen. As it constitutes most of the transcript, its optimization is critical for both mRNA stability and translation efficiency. A widely used approach to designing an optimal ORF and augmenting mRNA half-life is codon optimization, which refers to the substitution of rare codons with synonymous ones that are preferred by the translation machinery and pair with available tRNAs [29,30]. The 5′ and 3′ UTRs flanking the CDS serve as critical regulatory elements that are involved in ribosome recruitment and transcript stability, ultimately fine-tuning translational efficiency. The 5′ UTR is implicated in ribosome recruitment and initiation of the translation, ensuring the proper recognition of the start codon [31]. Additionally, the 5′ UTR can harbor internal ribosome entry sites (IRESs), allowing the direct recruitment of ribosomes in a cap-independent manner. 3′ UTR regulates transcript half-life and translation rates, while modulating immunogenicity. The incorporation of pyrimidine-rich sequences and motifs that are recognized by RNA-binding proteins (RBPs) dictates the stability and the rate of degradation of the transcripts. Because 3′ UTR length must be carefully optimized to balance transcript decay and translational efficiency, modern therapeutics frequently utilize tandem β-globin 3′ UTR sequences, significantly enhancing both mRNA longevity and protein expression across a broad range of cell types [30]. Moreover, antigen expression levels depend on the optimum combination of the UTRs [32]. Through a systematic screening process, Ye et al. discovered that pairing a Ces1d 5′ UTR with an AP3B1 3′ UTR increased the effectiveness of the broad-spectrum severe acute respiratory syndrome coronavirus 2 (SARS-CoV-2) vaccine (mCSA), as the secondary structures of these regions facilitated the ribosome recruitment to the mRNAs [33].

The 5′ cap constitutes another critical regulatory element. Structurally, the 5′ cap is defined by an N-7 methylated guanosine (m7G) attached to the 5′ end of the nascent mRNA through an inverted 5′-5′ triphosphate bridge (ppp). This structure is referred to as “Cap 0” and shields mRNA from 5′ → 3′ exonucleolytic degradation, while serving as a recognition site for the eukaryotic translation initiation factor 4E (eIF4E) [27]. Despite being the most observed cap, mRNAs harboring a “Cap 0” structure can be recognized as non-self RNA by pattern recognition receptors (PRRs), inducing an immune response and suppressing translation [34]. As a result, cap engineering focuses on the development of alternative Cap structures, such as “Cap 1” and “Cap 2”, which include an Nm on the ribose of the first and second nucleotide, respectively, evading immune activation and enhancing protein production [35]. Furthermore, a plethora of cap analogs has been engineered to enhance cap efficiency and minimize immune activation. The anti-reverse cap analogs (ARCAs) differ at the 3′ end, where the 3′-OH group of m7G has been substituted with 3′-O-Me, preventing reverse incorporation and promoting translation. Notably, recent approaches, such as trinucleotide analogs, nicotinamide adenine dinucleotide (NAD^+^) and flavin adenine dinucleotide (FAD) caps, conditional caps (photocleavable and pH-labile linkers) and caps linked with conjugates, expand the potential of mRNA therapeutics in immunotherapy and targeted delivery and enhance stability [30,36].

**Figure 2:**
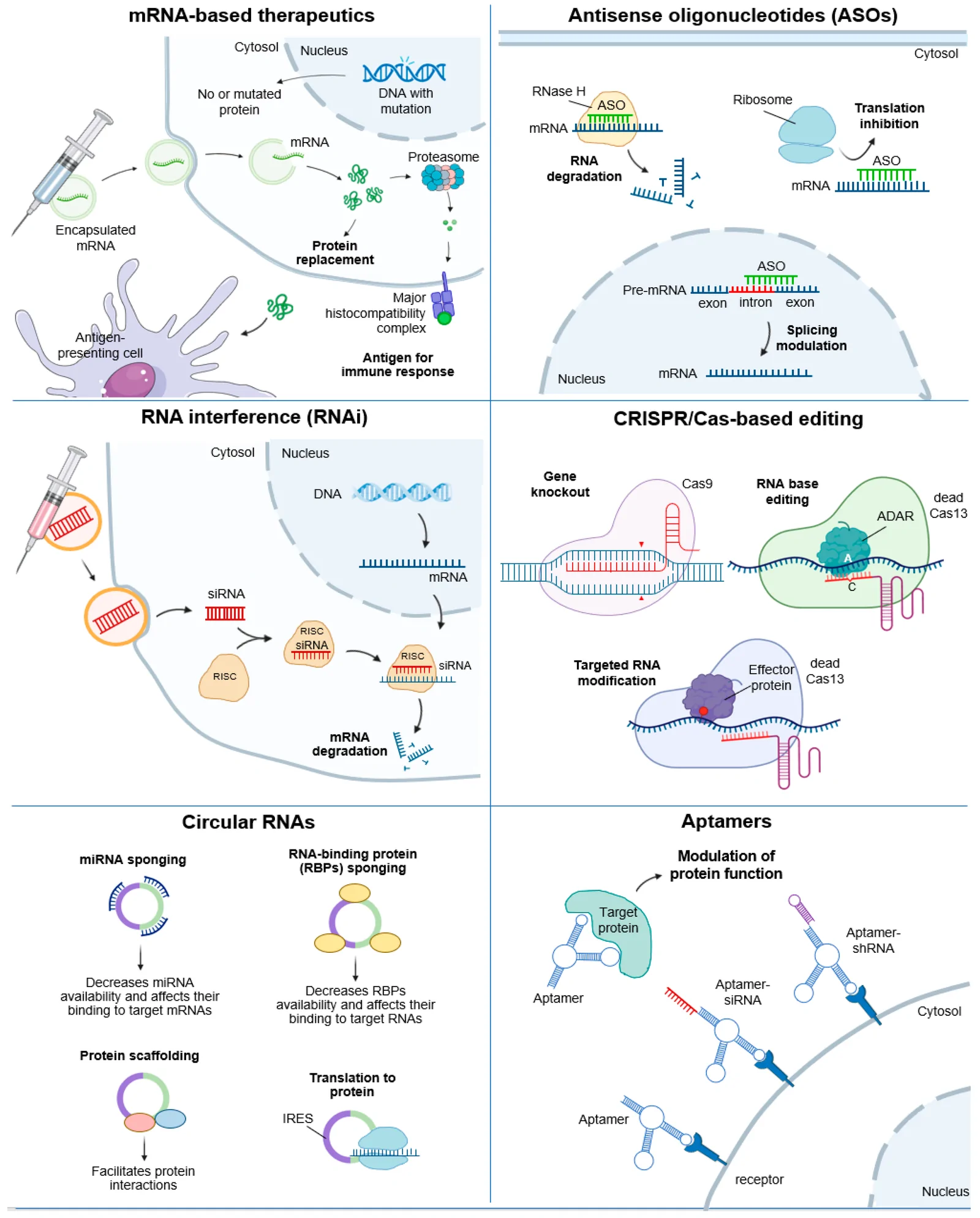
Schematic presentation of the major classes of RNA therapeutics and their mechanism of action.

The field of mRNA-based therapeutics relies on the delivery of a synthetic mRNA molecule and its subsequent translation by the cellular mechanism. The encoded protein may execute a specific function or constitute an antigen of either a pathogen or a tumor cell that activates a cell-mediated or antibody-mediated immune response (Figure 2) [37].

Based on those principles, mRNA platforms are being utilized in a broad range of applications, such as infectious diseases and antitumor vaccines, protein replacement therapies, cytokine-based immunotherapies, cell-based therapies and gene editing approaches. For example, mRNA vaccines for infectious diseases are designed for preventive action, as they induce an immune response for a pathogen-derived protein prior to infection [38]. On the contrary, cancer vaccines are used for disease treatment. By encoding tumor-associated and tumor-specific antigens, they induce antibody-mediated and cell-mediated immune responses that suppress further growth of the tumor and lead to regression of the cancer [39]. Moreover, mRNA molecules are evaluated as a tool for protein replacement therapies to encode and restore deficient proteins. This strategy provides notable benefits compared to protein- or DNA-based approaches, with the main one concerning safety, as it does not integrate into the genome and exhibits temporary action [40]. Regarding cytokine immunotherapy, the mRNA molecules are responsible for producing one or multiple immune-modulating cytokines directly at the tumor, overcoming the hurdle of generalized toxicity, while strengthening antitumor immune responses and counteracting the tumor’s immunosuppressive microenvironment [41]. In most cases, mRNA is inserted into the patient’s T cells and encodes the chimeric antigen receptor (CAR), ensuring its transient and regulated expression [42]. Synthetically, mRNA allows for rapid, cell-free production, completely avoiding the laborious scale-up associated with recombinant proteins. Functionally, mRNA overcomes the cellular delivery barriers of large protein therapeutics by utilizing the host’s endogenous translation machinery to express therapeutic targets directly inside the cell [18].

However, mRNA approaches still pose limitations, which mainly concern mRNA’s effective and targeted delivery, the unintended effects on off-target sites, its stability, as it is prone to degradation, its ability to activate the immune system, and its production challenges [19]. Due to its increased molecular weight, negative charge, and decreased stability, mRNA requires delivery platforms to prevent degradation, facilitate endosomal escape, and ensure targeted intracellular entry. To overcome these challenges, recent advances in delivery platforms, which utilize a diverse array of vectors, have significantly improved therapeutic stability, specificity, and safety, particularly for vaccines. While each platform poses limitations, the future of RNA therapeutics lies in engineering hybrid systems that merge their respective advantages to maximize delivery efficiency [41,43]. Furthermore, to overcome the intrinsic instability and immunogenicity of those molecules, while enhancing their translation, the synthetic structure of the mRNA is being optimized [41]. To obtain optimally designed IVT mRNA, even though machine learning (ML) and computational models offer useful solutions for sequence design, their efficacy is critically impeded by data scarcity and the inadequate RNA structure prediction tools for long sequences [29]. Ultimately, integrating biological insights, ML, and experimental validation remains crucial to surmounting these obstacles and fully exploiting the potential of computational mRNA optimization.

### 2.2. Antisense Oligonucleotides: Mechanistic Diversity in Gene Expression and Splicing Regulation

Antisense oligonucleotides (ASOs) are short, single-stranded, synthetic oligonucleotides that hybridize to cDNA or RNA sequences through Watson–Crick base pairing [44]. ASOs regulate gene expression by interacting with RNA and thus altering protein levels through modulation of RNA maturation, translation or degradation. The impact they exert is temporary, and they do not cause lasting alteration to the genome, rendering their administration a reversible approach to controlling gene expression [45,46]. The mechanism relies on either steric hindrance or, most commonly, degradation by the enzyme RNase H. More specifically, ASOs can sterically obstruct mRNAs through binding to regions such as splice junctions, changing splicing outcomes, or translation initiation sites, preventing translation from commencing. This approach is implemented in disorders driven by gain-of-function mutations with toxic effects or overexpression of specific proteins [47]. While both ASOs and siRNAs target specific RNA sequences and are employed for gene silencing, they differ in structure and mechanism of action. ASOs consist only of the strand that is complementary to the target, in contrast to siRNAs that are double-stranded, encompassing a sense and an antisense strand. The latter one is complementary to the RNA and guides the endonuclease Argonaute 2 (AGO2) of the RNA-induced silencing complex (RISC) to the target [48].

Undoubtedly, the therapeutic effectiveness of ASOs significantly depends on their structural and chemical design. The nucleotide sequence should be compatible with the target, enable potent binding to it and ensure the stability of the formed duplex. Regarding the target, the coding sequence is usually preferred, as its accessibility favors ASO binding. Alternatively, the regulatory elements of the 5′ and 3′ UTRs also pose a favorable target [46]. Furthermore, ASOs are chemically modified to overcome their inherent instability and inadequate cellular uptake, improving their resistance to degradation, enhancing their binding to the target, prolonging their activity and diminishing their toxic effects [46]. The first generation of chemical modifications concerns the phosphate backbone of the ASOs. Those alterations are crucial, as their negative charge limits cellular uptake and hinders the interactions with RNA targets. In particular, the oxygen atoms of the phosphodiester bond that do not participate in the bond formation are substituted by a sulfur, methyl or amine group. The phosphorothioates (PS), being the most abundant modification, not only increase the half-life and the stability of the molecules, but they also promote RNase H activity, rendering the mechanism more effective [49]. This does not apply to the methylphosphonates, which are less common. Their advantage lies in the neutralization of the negative charge of the backbone that increases stability but reduces solubility and poses a hurdle in the permeability of the cellular membrane [50]. Apart from modifying the backbone, structural alterations of the pentose sugar are also extensively studied, aiming to reinforce in vivo performance [51,52,53]. Of note, the second generation of chemical modifications features alkyl alterations at the 2′ position of the pentose, generating mainly 2′-O-methyl (2′-OME) and 2′-O-methoxyethyl (2′-MOE) nucleotides, which are usually incorporated along with a PS backbone. While these modifications enhance binding affinity and minimize toxicity and immune activation, they do not recruit RNase H and, therefore, render the ASOs less effective in inducing degradation. Lastly, the third generation of modifications is applied to the furanose ring, producing nucleotide analogs with enhanced binding affinity, resistance to nucleases and improved pharmacokinetic properties [54]. They include locked nucleic acids (LNAs), phosphorodiamidate morpholino oligomers (PMOs), and peptide nucleic acids (PNAs) [55].

In general, ASOs regulate gene expression through three main mechanisms of action: (1) degradation of target RNA, (2) steric blockage of important mRNA regions and (3) modulation of pre-mRNA splicing (Figure 2). Notably, they function both in the cytoplasm and the nucleus, where they intervene in the mRNA maturation processes. Primarily, transcript degradation is accomplished through multiple pathways. Specifically, the main application of ASOs relies on RNase H-mediated degradation. This mechanism utilizes a class of ASOs termed as gapmers, which are single-stranded oligonucleotides composed of a central DNA region interspersed with sugar-modified ribonucleotides that are resistant to degradation by RNase H [56]. Upon hybridization with the target mRNA, gapmers form DNA-RNA heteroduplexes that are recognized by RNase H, leading to selective cleavage and destruction of the RNA strand, while also conferring resistance to exonuclease-mediated degradation [46,57]. Furthermore, hybridization on the poly(A) tail results in shortening of this region and thus destabilization and degradation of the transcript. The RNAi pathway may also be induced, as ASOs can be loaded to the RISC and bind to complementary targets, resulting in either translation repression or mRNA degradation. Furthermore, hybridization of ASOs to pre-mRNAs promotes exon skipping and may generate transcripts with premature stop codons, which are subsequently targeted for degradation through the nonsense-mediated mRNA decay (NMD) pathway [46,58]. More recently, a novel mechanism has been developed, in which RNase H-inactive ASOs direct mRNAs to lysosomal degradation pathways using autophagosome-tethering compound (ATTEC) technology, enabling efficient cytoplasmic mRNA knockdown through an RNase H-independent manner [59].

Another class of ASOs, known as steric-blocking ASOs, exert their effects through steric obstruction without inducing transcript degradation. These oligonucleotides are designed to bind target RNAs with high affinity and, as they do not recruit RNase H, they do not promote cleavage of the RNA [60]. This mechanism is exemplified by PMOs, which modulate gene expression by hybridizing with target transcripts and preventing either the assembly of the translation machinery or the access to splice junctions. Additionally, ASOs can also modulate RNA function in diverse ways to either repress or enhance protein expression. Of note, they may disrupt inhibitory RNA structures, impede upstream initiation codons, or interfere with microRNAs (miRNAs), thereby promoting translation [61]. Conversely, binding to the 5′ UTR or initiation codon can prevent translation initiation. ASOs can further influence RNA stability, localization, and nuclear export by altering RNA–protein or RNA–miRNA interactions. Interestingly, they can also regulate RNA maturation by modulating splicing or polyadenylation patterns. Overall, these mechanisms enable precise gene expression modulation, while preserving transcript integrity and, therefore, alleviate the limitations of RNase H-dependent strategies for challenging therapeutic targets [62].

Programmable regulation of alternative splicing has long been a central focus in RNA biology, offering significant potential for correcting splicing defects in disease and for elucidating splicing mechanisms. Steric-blocking antisense oligonucleotides, also referred to as splice-switching oligonucleotides (SSOs), are synthesized to modulate RNA splicing by hybridizing to specific sequences within a pre-mRNA target, such as 5′ or 3′ splice sites, exonic splicing enhancers (ESEs), or exonic splicing silencers (ESSs). By occupying these regulatory regions, SSOs hinder the access of spliceosomal components and associated splicing factors, thus altering splice site selection, influencing exon inclusion or exclusion in the mature mRNA and modifying the protein repertoire of the cell. Binding to splicing enhancers impairs the recruitment of splice-promoting factors, leading to exon skipping, while targeting splicing silencers hinders repressor protein binding, allowing for exon inclusion [63,64]. Through these mechanisms, ASO-mediated splice modulation can either restore gene expression or intentionally dysregulate it, enabling the correction of pathogenic splicing events caused by mutations, the circumvention of deleterious mutations, or the generation of alternative protein isoforms [65].

Despite their growing commercial acceptance, the incorporation of ASOs in therapeutics is constrained by bottlenecks in bioavailability, safety, clinical assessment strategies, and, primarily, delivery, requiring innovative solutions [66]. As mentioned, oligonucleotides are subjected to chemical modifications, which enhance their stability, functionality, and pharmacokinetic properties, while being responsible for their off-target activity. An additional limitation concerns their penetration across physiological barriers, which is tackled by their chemical conjugation with transporter molecules, while simultaneously increasing their specificity [67]. Additionally, apart from chemical modifications and careful structural manufacturing, in silico sequence design using ML platforms, such as ASOptimizer and eSkip-Finder, enables the simultaneous prediction of on-target efficacy and off-target cytotoxicity, achieving an optimal balance between efficacy and toxicity [68,69]. Ultimately, overcoming these challenges requires further research, integrating advanced delivery platforms, chemical optimization, and improved clinical approaches.

### 2.3. Mechanisms and Therapeutic Potential of RNAi-Mediated Gene Silencing

Our understanding of gene silencing was fundamentally altered in 1998, when Fire and Mello released their seminal study demonstrating that double-stranded RNA (dsRNA), rather than single-stranded RNA (ssRNA), is the trigger for RNAi in *Caenorhabditis elegans* [70]. This work provided the crucial mechanistic framework required to unify disparate observations of gene silencing, including co-suppression in plants, quelling in fungi and sense or antisense RNA in animals [71]. RNAi constitutes a conserved cellular process that mediates post-transcriptional gene silencing and can be harnessed for therapeutic applications. Apart from its role in endogenous gene regulation, RNAi has an essential role in anti-viral immunity and maintenance of genomic integrity against transposable elements [72]. This pathway is activated by small RNA molecules, including siRNAs, endogenous miRNAs, PIWI-interacting RNAs (piRNAs), and promoter-driven short hairpin RNAs (shRNAs), all of which guide sequence-specific suppression of target gene expression. Therefore, RNAi-based therapeutics exploit these endogenous mechanisms to achieve precise gene expression regulation [73,74].

The RNAi pathway depends on DICER, a cytoplasmic RNase III enzyme that is essential for the processing of dsRNA precursors. These precursors, whether endogenously derived or exogenously introduced as stem-loop or shRNAs, are cleaved into siRNAs or miRNAs. These small RNAs are subsequently loaded into the RISC, which constitutes a ribonucleoprotein (RNP) complex. Within this assembly, an Argonaute (AGO) protein acts as the catalytic component, while the small RNA molecule acts as a guide to identify target mRNAs through Watson–Crick base pairing [62]. Notably, RNAi machinery exhibits remarkable effectiveness in human cells, due to the ability of a single RISC to execute multiple rounds of sequence-specific mRNA cleavage [75].

In general, two distinct classes of small non-coding RNAs predominantly participate in RNAi: miRNAs, which are recognized as endogenous molecules responsible for gene expression regulation, and siRNAs, which function as crucial aspects in maintaining genome integrity and defending against viruses and transposable elements [76]. Although both comprise essential components of the RNAi machinery, their function and targeting modalities differ significantly. Regarding miRNAs, they are endogenously encoded and typically span 18–25 nucleotides in length. They are a critical component of the miRNA-induced silencing complex (miRISC) and usually recognize sequences within the 3′ UTR of target mRNAs through partial complementarity with the seed region of their sequence. Of note, they possess the ability to modulate cellular pathways by simultaneously targeting multiple mRNA transcripts. Their regulatory effects primarily result in translational repression, deadenylation and subsequent mRNA degradation [77]. Conversely, siRNAs have emerged as indispensable tools for sequence-specific gene expression regulation. While endogenous siRNAs exist, their research relies mainly on chemically synthesized siRNAs that are delivered into the cells to selectively regulate the expression of a single target gene. These molecules are designed to exhibit high specificity for target mRNA sequences, as the siRNA-induced silencing complex (siRISC) requires perfect complementarity, more commonly to the CDS, to trigger site-specific cleavage and degradation of the transcript (Figure 2) [74,78].

In comparison to traditional therapeutic approaches involving small molecules, miRNAs and siRNAs are distinguished as they can be engineered to target any gene and provide the advantage of selectively targeting transcripts of undruggable proteins [79]. miRNA-based therapeutics generally follow two distinct paradigms. The first one concerns miRNA inhibition, which employs single-stranded oligonucleotides, such as antagomirs or anti-miRNAs, that prevent the function of miRNAs [80]. On the contrary, the miRNA replacement approach utilizes miRNA mimics to restore the function of decreased endogenous miRNAs, eventually leading to gene silencing [81]. Regarding therapeutic approaches that include siRNAs, they rely on the exogenous induction of the RNAi pathway to achieve gene silencing of a specific target of interest. A primary advantage of this approach, when compared with small-molecule inhibitors or monoclonal antibodies, which target three-dimensional entities, pertains to the precise Watson–Crick base pairing with the primary nucleotide sequence of the target. By overcoming the requirement for high-affinity protein binding, siRNA platforms offer a broader therapeutic reach [79,82].

Notwithstanding the inherent advantages of siRNA-based therapeutics, their development has been hindered by significant obstacles, including decreased stability and rapid degradation of unmodified siRNAs by nucleases, as well as immunogenicity and straightforward clearance due to their low molecular weight [83]. Furthermore, in vivo siRNA activity is significantly constrained by administration route selection, and endosomal entrapment. These challenges, compounded by the potential of system toxicity and off-target effects, are being increasingly mitigated through the incorporation of chemical modifications to stabilize siRNA and minimize protein binding, nanoparticle encapsulation to shield them and restrict non-specific uptake, and tissue-targeting ligands to refine delivery precision. Ultimately, achieving therapeutic efficacy depends on siRNA escaping endosomes through membrane fusion or disruption, a challenge addressed using pH-sensitive liposomes, cell-penetrating peptides, endosomal disruptors, and advanced nanoparticles [84].

### 2.4. Functional Diversity in Advanced RNA Therapeutics: CRISPR/Cas, circRNAs and Aptamers

The landscape of RNA therapeutics is rapidly expanding, transitioning from classical approaches that include coding and gene silencing molecules, to a varied array of advanced RNA modalities with more diverse functional entities. The new generation of RNA therapeutics introduces unique structural and functional systems, circumventing obstacles posed by older technologies and providing new capabilities. Recent modalities include CRISPR/Cas guide RNAs (gRNAs) that enable targeted genome engineering, circRNAs that provide an advanced, exonuclease-resistant therapeutic platform, along with aptamers that extend the utility and structure of RNA by functioning as antibodies (Figure 2).

Originally adapted from the physiological adaptive immune system of prokaryotic organisms, the CRISPR/Cas platforms are utilized for precise genome editing of mammalian cells, introducing genomic alterations. The mechanism relies on an RNA-guided Cas nuclease and a gRNA that directs the enzyme to a specific locus through sequence complementarity with the target. Upon binding, the Cas effector executes site-specific cleavage of either double-stranded DNA (dsDNA) or ssRNA, inducing gene knockout or targeted sequence modification [85]. Beyond the foundational applications of Cas9-mediated genome editing, the CRISPR applications have diversified to include effectors, such as Cas12a and Cas13, acting on DNA and RNA, respectively [86,87]. The therapeutic potential of the CRISPR/Cas genome-editing approach lies in its capacity to introduce permanent genetic alterations, offering a transformative paradigm for the treatment of previously challenging-to-treat health conditions, such as genetic disorders, cancer and infectious diseases [88]. Interestingly, therapeutic strategies that target directly the mRNA level, such as RNA-targeting CRISPR/Cas13 systems or endogenous adenosine deaminase acting on RNA (ADAR) recruitment, are also characterized by immense clinical potential. Acting entirely within the cytoplasm, these transcriptome-editing approaches bypass the need for nuclear import and do not induce permanent double-strand DNA breaks. This ensures rapid, transient and fully reversible modulation of gene expression, completely avoiding the risks of genomic toxicity and mutagenesis associated with DNA-level interventions [47,89]. Despite its profound curative promise, CRISPR editing is constrained by safety, delivery, and scalability barriers. Advancing the field demands broader genomic coverage, optimized gRNA design, and tools capable of accurately identifying multigene transcripts. In silico modeling is increasingly employed to engineer gRNAs and promote on-target cleavage, while avoiding off-target effects. These algorithms rely either on sequence-only pipelines that screen reference genomes for structural similarities, or on algorithmic scoring metrics that grade potential on-target and off-target sites [90]. Notably, a critical obstacle concerns the preexisting immunity against the CRISPR effector proteins. Even though computational tools can efficiently predict immunogenicity and identify epitopes for silencing, more robust models are needed to enable clinical incorporation. Current strategies to mitigate host immunity include epitope shielding, use of effector protein orthologs, localized delivery to immune-privileged tissues, transient immunosuppression, and regulatory T-cell therapy [91]. Additionally, adverse effects beyond indels have been observed and include extensive deletions, truncations, chromothripsis, and translocations between chromosomes. Consequently, the development of diagnostic frameworks that track these structural alterations is urgent. As editing accuracy is determined by patient-specific factors, along with numerous additional variables, risk evaluation should incorporate both personalized off-target risk profiling and a comprehensive risk–benefit assessment [92].

While linear mRNAs are constrained by increased instability, temporary expression and immunogenicity, circRNAs constitute a more durable alternative. Synthesized through a noncanonical back-splicing mechanism, they constitute covalently closed ssRNAs that are deprived of 5′ and 3′ ends, rendering them inherently resistant to exonuclease-mediated degradation [93]. Historically regarded as splicing artifacts, circRNAs have recently emerged as conserved molecules with multiple regulatory roles, acting as molecular “sponges” for miRNAs, modulating the efficiency of mRNA translation, and serving as structural scaffolds for RNP complexes. circRNAs can facilitate cap-independent translation through IRESs, as well as N6-methyladenosine (m6A)-mediated translation. Furthermore, their disease-specific expression profiles render them promising diagnostic biomarkers [94,95]. By providing sustained protein synthesis and robust non-coding functionality, circRNAs represent a durable alternative to linear mRNA for diverse therapeutic interventions. Synthetic circRNAs have emerged as an efficient alternative for both therapeutics and vaccine development. Currently, circRNA-based approaches are being evaluated across a broad spectrum of pathological conditions, including viral infections, oncology, neurological injuries and metabolic disorders, rendering circRNAs as versatile preventive and therapeutic agents [94]. In the context of vaccine development, the primary objective is to evoke immunological memory through the generation of B and T memory cells [95]. Recent evidence confirms that circRNA vaccines effectively induce adaptive immune responses. Beyond viral vaccines, this platform shows considerable promise in presenting tumor neoantigens for cancer immunotherapy and modulating dysfunctional autoimmune responses [17]. Interestingly, elevated protein yields and prolonged expression levels make circRNAs a compelling alternative to linear mRNA for protein replacement therapies. Owing to their reduced immunogenicity and extended translational rates, circRNAs represent a more suitable platform compared to mRNA for gene editing applications. Subsequently, circRNAs are poised to become a key component of personalized medicine, offering an efficient alternative to traditional RNA-based treatments. However, their sustained expression kinetics heighten the risk of increased off-target effects [96]. Their therapeutic promise is also constrained by sequence optimization, cyclization efficiency, and chemistry, manufacturing and control (CMC) processes. To address these hurdles, researchers have designed diverse circRNA overexpression vectors to achieve accurate, high-yield circularized products [97]. However, residual linear precursors are frequently present, inducing immune system activation and rendering post-cyclization purification essential. This can be achieved either through RNase R digestion, or by size exclusion methods, such as PAGE and high-performance liquid chromatography (HPLC) [94]. Furthermore, as they do not possess a 5′ cap, they rely on IRES for translation, which often correlates with decreased translation efficiency. To overcome this, translation can be enhanced by optimizing five core components: vector topology, the 5′ and 3′ UTRs, IRES sequences, and synthetic aptamers that directly recruit the translation machinery [98]. Finally, clinical safety requires alleviating off-target risks. Utilizing tissue-specific delivery systems is crucial for increasing vaccine efficacy [99]. Decreasing cryptic ORFs and unintended translation through predictive in silico scanning and proteomic validation, supported by systematic reporting of junction design, should also be incorporated in risk-mitigation processes [93].

Functioning as the nucleic acid-based analogs of monoclonal antibodies, aptamers are synthetic, ssDNA or RNA oligonucleotides distinguished by their high specificity and affinity for diverse molecular targets. They were initially engineered in 1990 through the systematic evolution of ligands by exponential enrichment (SELEX) methodology [100,101]. Upon cell internalization, these oligonucleotides fold into stable, complex three-dimensional conformations that permit direct structural interaction with their desired targets [102,103]. Their broad functional versatility makes them a highly attractive therapeutic platform. Similar to monoclonal antibodies, aptamers act as antagonists by binding to specific epitopes, thereby inhibiting the protein activity and disrupting protein–protein or receptor–ligand interactions. In addition, by recognizing cell-surface receptors, aptamers facilitate the cell-specific delivery and endocytosis of various agents, including drugs, siRNAs, shRNA, anti-miRNAs, miRNA mimics, ASOs, small molecules, and various nanoparticles [104]. Finally, aptamers can be readily conjugated to liposomes and other delivery platforms, facilitating the precise transfer of small molecules, proteins, nucleic acids, and entire CRISPR/Cas9 systems [105,106]. Of note, the activity of aptamers can be interrupted by the administration of complementary oligonucleotides, known as “antidotes” [107]. Aptamers represent an auspicious therapeutic class for conditions including ocular diseases and blood disorders. Their structural correlation to immunoglobulins renders them particularly effective for modulating complex disease pathways in oncology and autoimmune-mediated inflammatory conditions [108,109]. Their advantage over monoclonal antibodies lies in their ability to interact with otherwise undruggable proteins or regulate protein expression, while also being manufactured in an affordable manner [104]. However, critical obstacles, such as the innate immunogenicity of oligonucleotides and their susceptibility to degradation, have limited the therapeutic application of aptamers [110]. These challenges have been largely mitigated through advanced chemical engineering, enhancing their metabolic stability and enabling their use as naked conjugates [103]. An additional barrier to the clinical translation of aptamers is their adverse pharmacokinetics. Their limited size renders unmodified aptamers prone to prompt renal clearance and enzymatic degradation. Extending aptamer half-life through chemical modifications and conjugation may come at the expense of binding affinity, structural integrity, or safety, highlighting the requirement to balance stability with functionality. Moreover, receptor-mediated uptake platforms can partially overcome delivery constraints, especially regarding solid tumors [111]. Diagnostic aptamers face further challenges concerning target specificity and cross-reactivity, which must be mitigated with rigorous negative selection during the SELEX protocols. Finally, clinical safety remains a concern due to potential toxicities from high anionic charge, chemical alterations, or tissue deposition. Consequently, while toxicological data remains inadequate, advancing aptamer therapeutics requires optimized therapeutic approaches and appropriate routes of administration [112].

## 3. Overcoming the Delivery Bottleneck

RNA molecules, ranging from large single-stranded mRNAs to small double-stranded siRNAs, are polyanionic macromolecules that lack the necessary physicochemical properties for unassisted systemic transport, rendering them highly susceptible to enzymatic hydrolysis and unable to cross the hydrophobic lipid bilayer of target cells [113]. Consequently, despite the programmable versatility of RNA as a therapeutic target, its clinical utility is fundamentally dictated by the efficiency of its delivery. Thus, the “delivery bottleneck” refers to the multi-stage challenge of protecting this unstable molecule from the extracellular environment, navigating it to the target tissue and ensuring its functional release into the cytoplasm [114].

Successful intracellular delivery of RNA therapeutics requires overcoming a series of physiological and cellular barriers, starting with ribonucleases in the blood and interstitial fluids that degrade RNA within minutes of systemic injection. While chemical modifications enhance nuclease resistance, they are generally insufficient to ensure systemic stability on their own [115,116]. Additionally, the negative charge of the RNA backbone leads to non-specific interactions with serum proteins, a process known as opsonization, which can trigger the mononuclear phagocyte system (MPS) and, finally, trap and destroy the drug in the liver and spleen before it can reach its intended target [117,118]. For smaller RNAs, including siRNAs and ASOs, renal filtration is a significant hurdle. Molecules with a hydrodynamic radius smaller than ~10 nm are subjected to rapid renal elimination [119,120]. Conversely, larger delivery vehicles, such as the LNPs, must navigate the vascular endothelium. While the fenestrated liver endothelium, which has 50–300 nm diameter pores, permits nanoparticle passage, accessing tissues with specialized vascular barriers, such as the blood–brain barrier or solid tumors with high interstitial pressure, requires sophisticated engineering of the vehicle’s surface properties [121,122,123].

Upon arriving at the target site, the delivery vehicle must cross the plasma membrane, where electrostatic repulsion between the polyanionic therapeutic agent and the negatively charged cellular surface precludes spontaneous entry [124,125]. Most delivery systems rely on endocytosis (macropinocytosis, clathrin-mediated, or caveolae-mediated); however, therapeutic molecules often remain trapped within endosomes that undergo progressive maturation [126]. This represents the most critical obstacle in delivery, as RNA faces enzymatic degradation within the lysosomal compartment, unless it achieves endosomal escape, an inefficient process where typically only 1–2% of the internalized dose reaches the cytoplasm in a functional state [127,128].

### 3.1. LNPs as the Current Gold Standard for RNA Therapeutics

LNPs have emerged as the most successful platform for RNA delivery, serving as the vehicle for both siRNA delivery and the COVID-19 mRNA vaccines [129,130,131]. Unlike traditional liposomes, LNPs possess a solid core and a specialized architecture optimized for RNA protection and endosomal escape [132]. An engineered LNP typically features a four-component architecture, which consists of distinct lipid types that each serve a specific structural or functional role. The most critical component is the ionizable lipid, which remains neutral at physiological pH (~7.4) to minimize toxicity and extend circulation time, while ultimately enhancing transfection efficiency. However, these lipids undergo protonation within the acidic endosomal environment (pH ≈ 5.0–6.0), triggering a transition to a hexagonal phase that disrupts the endosomal membrane and facilitates the release of the RNA into the cytoplasm [133,134]. To ensure stability, polyethylene glycol-modified lipids (PEG lipids) provide a “stealth” layer on the nanoparticle surface, preventing aggregation during manufacturing and reducing non-specific protein binding in the blood (Table 1) [135].

Furthermore, cholesterol acts as a structural stabilizer by occupying interstitial spaces within the lipid matrix. By filling these molecular “gaps,” the packing density is enhanced, and the fluidity of the nanoparticle is modulated, ensuring it is neither too stiff nor too liquid for transit [139,140]. Finally, helper lipids, such as distearoylphosphatidylcholine (DSPC), provide structural integrity and assist the LNP in transitioning from a stable sphere into a state that can easily fuse with cellular membranes, allowing the RNA to successfully “escape” the endosome and reach its destination [141].

Beyond their chemical composition, the clinical success of LNPs is also a result of precision engineering at the manufacturing level. LNP success is driven by a shift in manufacturing from traditional bulk mixing to impingement jet mixing. Bulk mixing involves the macro-scale stirring of large volumes of lipids and RNA; however, this method often lacks the speed and precision required for uniform assembly, leading to high polydispersity. In contrast, impingement jet mixing is a high-efficiency, passive, rapid-mixing technique where the ethanolic lipid phase and the aqueous RNA phase collide at high velocities within a small, specialized chamber. By operating at high Reynolds numbers (>10,000), impingement jet mixing creates intense turbulence that ensures near-instantaneous, homogeneous mixing [142,143]. This precision allows engineers to produce particles with a highly uniform size, typically between 60 and 100 nm, ensuring consistent pharmacokinetic profiles and reproducible therapeutic performance across manufacturing batches [144].

### 3.2. Polymer-Based and Peptide-Based Nanocarriers

While LNPs currently represent the gold standard for RNA delivery, polymeric and peptide-based nanocarriers have emerged as essential alternatives due to their superior structural stability and the high degree of chemical flexibility they offer for tissue-specific targeting [10,145]. Unlike lipid-based systems, which can be limited by their tendency to accumulate in the liver, polymeric and peptide-based nanocarriers allow for precise functionalization to navigate diverse physiological environments [146]. Cationic polymers, such as polyethylenimine, have long served as a benchmark in this field because they complex spontaneously with the negatively charged phosphate backbone of RNA through electrostatic interactions to form stable polyplexes [147]. More precisely, the primary mechanism of intracellular release for these systems is the “proton sponge effect,” which relies on the high buffering capacity of the polymer’s amine groups. As the endosome matures and its internal pH drops, the polymer sequesters incoming protons. To maintain electrical and osmotic neutrality, a subsequent influx of chloride ions and water occurs, leading to osmotic swelling and the eventual physical rupture of the endosomal membrane [148,149].

Despite the efficiency of this mechanism, the high charge density of traditional cationic polymers is frequently associated with significant cytotoxicity and non-specific interactions with serum proteins. To address these limitations, modern engineering has shifted toward the development of neutral and self-assembled polymers [150]. Neutral polymers, such as poly(lactic-co-glycolic acid) (PLGA), offer enhanced biocompatibility and have been successfully applied to deliver mRNA encoding antigenic proteins for cancer immunotherapy [151,152]. These nanoparticles provide a prolonged and controlled release of the therapeutic agent, which lengthens the exposure time of antigens internally and significantly enhances the resulting immune response [153,154]. Furthermore, self-assembled polymers provide high structural tunability, enabling precise adjustments to particle size, surface charge, and ligand modifications to facilitate targeted delivery to specific tissues [155]. More specifically, one approach involves synthesizing poly(disulfide)s through the photocrosslinking of modified thioctic acid monomers, including zinc-coordinated diacetamide and guanidine [156]. The resulting polydisulfide backbone is highly sensitive to reducing environments, facilitating a “smart” controlled release of the encapsulated RNA once it reaches the cytoplasm [10].

Beyond linear and self-assembled polymers, dendrimers have emerged as promising three-dimensional carriers for nucleic acid delivery, offering a unique architectural advantage due to their highly branched, tree-like structure. In this architecture, each new layer of branches is referred to as a “generation”; as the tree adds more generations, it develops a more complex branching structure with an increasing number of surface binding sites, which allows it to hold the RNA more effectively. Specifically, poly(amidoamine) (PAMAM) dendrimers use these positively charged surface points to bind to the negatively charged RNA, wrapping it into a stable, protective package [157,158]. Once inside the cell, the internal structure of the dendrimer acts like a sponge for protons (H^+^); this causes the endosome to swell and burst, successfully releasing the RNA into the cytoplasm. The precise, layer-by-layer synthesis of these molecules ensures strict control over molecular weight and binding affinity, allowing for highly reproducible delivery profiles. To mitigate the inherent toxicity of high charge density, surface-engineering strategies such as PEGylation are utilized to shield the cationic exterior, which successfully reduces cytotoxicity and prevents non-specific protein binding in systemic circulation [159]. Furthermore, the attachment of specific ligands to the peripheral branches enables active, tumor-specific targeting and improved biodistribution. These engineered platforms have demonstrated notable efficacy in inducing protective immunity against infectious diseases, highlighting a modular versatility that extends the utility of dendrimers far beyond oncological applications [160].

Parallel to polymeric advancements, peptide-based systems, including cell-penetrating peptides (CPPs), offer a biologically inspired approach to crossing cellular membranes [161,162]. CPPs, such as the human immunodeficiency virus type 1 (HIV-1) derived TAT peptide or synthetic polyarginine, interact strongly with the negatively charged cell surface to facilitate internalization through direct membrane translocation or active endocytic pathways. To overcome the lack of specificity in these early-generation peptides, recent studies focus on “activatable” CPPs (aCPPs) [163]. These systems utilize a polyanionic domain to shield the cationic peptide during transit, only releasing it when specific local triggers are encountered, such as the acidic pH of a tumor microenvironment or specific extracellular proteases [164]. Undoubtedly, the integration of these polymeric and peptide-based strategies establishes a more versatile and robust toolkit for RNA therapeutics that extends well beyond the current capabilities of lipid-based platforms.

### 3.3. Ligand–RNA Conjugates

In the context of precision medicine, ligand–RNA conjugates represent the most direct path to tissue-specific targeting by bypassing the structural complexity of nanoparticle carriers. The pre-eminent example of this approach is the conjugation of N-acetylgalactosamine (GalNAc) to siRNA, which has revolutionized the treatment of hepatic diseases [165]. This system exploits the natural pathway of desialylated glycoproteins by targeting the asialoglycoprotein receptor (ASGPR), a high-capacity, C-type lectin expressed almost exclusively on the sinusoidal surface of hepatocytes [166]. To achieve peak binding affinity, synthetic conjugates typically utilize a triantennary GalNAc structure, comprising three sugar units arranged to match the precise spatial orientation of the ASGPR binding sites. This interaction results in sub-nanomolar affinity, ensuring that the siRNA localizes directly to the liver with incredible precision upon systemic administration [167]. Once the conjugate binds to the receptor, the entire complex is rapidly internalized through clathrin-mediated endocytosis. A critical feature of this delivery cycle is the behavior of the complex within the intracellular environment. Upon reaching the acidic environment of the early endosome (pH ~5–6), the change in pH triggers the dissociation of the GalNAc-siRNA conjugate from the ASGPR. While the siRNA is released to engage the cellular RNAi machinery in the cytoplasm, the receptor is not degraded; instead, it is recycled back to the cell surface [168]. This continuous recycling allows a single hepatocyte to internalize a vast quantity of therapeutic RNA over time, contributing to the high potency observed in clinical settings. It should be noted that the siRNA molecule is directly exposed to the extracellular environment due to the lack of the protective shell of a lipid nanoparticle or polymer in these conjugates. Consequently, success depends on extensive chemical engineering of the RNA backbone. The siRNA is typically modified with phosphorothioate linkages to resist nuclease degradation and 2′-modifications to enhance stability and reduce immunogenicity [169]. These modifications, combined with the efficient targeting of GalNAc, allow for drugs such as givosiran and inclisiran to be administered subcutaneously [137].

Antibody–oligonucleotide conjugates (AOCs) represent a burgeoning frontier designed to reach extrahepatic tissues with high specificity. This approach leverages the high affinity of monoclonal antibodies for cell-surface receptors that are preferentially expressed in target organs, most notably the transferrin receptor 1 (TfR1) found in skeletal muscle and cardiac tissue. By exploiting the natural recycling pathway of TfR1, these conjugates are internalized through receptor-mediated endocytosis, allowing the oligonucleotide cargo to bypass the physiological barriers that typically limit uptake in nonhepatic tissues [170,171]. The functional performance of an AOC is dictated by the precise engineering of the linker chemistry and the drug-to-antibody ratio (DAR), which must balance systemic stability with the efficient intracellular release of the RNA payload [172]. Furthermore, because the RNA is directly conjugated to the antibody without the protection of a nanocarrier shell, success relies on extensive chemical modifications, such as Nm and 2′-fluoro substitutions, to resist nuclease degradation and prevent immunogenicity [173].

### 3.4. Viral vs. Non-Viral Vectors

The development of gene therapeutics requires a strategic choice between viral and non-viral delivery systems, a decision primarily dictated by the trade-off between transduction efficiency and safety. Viral vectors, such as adeno-associated viruses (AAVs) and lentiviruses, have evolved to bypass cellular barriers, resulting in extremely high delivery efficiency [174]. However, these biological systems are constrained by limited capacity, approximately 4.7 kb for AAV, and high immunogenicity, which often prevents repeatable dosing due to the development of neutralizing antibodies [175]. Furthermore, viral manufacturing remains complex, expensive, and difficult to scale, while certain vectors such as lentiviruses carry the inherent risk of insertional mutagenesis.

In contrast, non-viral vectors, including LNPs and polymers, offer a virtually unlimited capacity and a significantly lower immunogenic profile, making them ideal for chronic treatments and N-of-1 therapies [176]. While their efficiency was historically moderate compared to viral counterparts, ongoing engineering advancements have significantly improved their performance. Because non-viral systems rely on chemical synthesis, they are highly scalable and provide a safer, transient expression profile that avoids the risks of permanent genomic alteration [177]. Consequently, the scalability and low immunogenicity of these vectors have positioned them as the preferred engineering choice for the next generation of RNA drugs.

Despite these advantages, the clinical application of many RNA therapeutics is currently constrained by “liver-centricity,” as the liver serves as the natural clearance organ for most nanoparticles. Achieving extrahepatic tropism—delivery to the lungs, brain, or immune cells—is considered the final piece of the delivery puzzle. A prominent strategy to resolve this bottleneck is selective organ targeting (SORT), which involves the addition of a specialized fifth “SORT lipid” to the standard four-component LNP architecture [178]. By modulating the internal composition and surface chemistry of the nanoparticle, these lipids shift accumulation from the liver to specific organs like the lungs or spleen, effectively re-engineering the biodistribution profile of the therapeutic payload.

The resolution of these delivery bottlenecks is increasingly being accelerated through the integration of AI and high-throughput screening. These computational tools enable the rapid evaluation of thousands of lipid combinations to identify unique formulations capable of crossing the blood–brain barrier or selectively entering tumor cells. This shift toward AI-driven precision engineering is resolving the constraints that have limited the field for decades, ultimately allowing RNA therapeutics to reach previously inaccessible clinical targets [179,180].

From a regulatory perspective, securing FDA and European Medicines Agency (EMA) approval for non-viral delivery systems remains heavily constrained by toxicological and structural hurdles. For LNPs, a primary challenge is the potential for dose-dependent immunogenicity, transient hepatotoxicity, and the activation of the complement system (complement-activation-related pseudoallergy, or CARPA), which can lead to severe hypersensitivity reactions [181]. Polymeric and peptide vectors face even steeper translational barriers as their high charge densities, while effective for cellular uptake, frequently cause significant membrane disruption and systematic cytotoxicity in vivo. Additionally, ensuring long-term batch-to-batch structural uniformity, controlling polydispersity, and validating the in vivo biodegradation kinetics of synthetic polymers present complex CMC challenges that often stall clinical progress [182]. Consequently, the regulatory landscape demands rigid characterization of vehicle-associated toxicity profiles independently from the RNA payload before these delivery strategies can achieve widespread clinical approval.

## 4. Clinical Applications and Therapeutic Areas

Over the past decades, research has led to the emergence of mRNA vaccines as a novel platform in vaccinology. The first human trial of an mRNA vaccine for an infectious disease was conducted in 2013 for protection against rabies virus (NCT02241135). The global outbreak of COVID-19 served as a critical catalyst for biotechnological innovation, drastically accelerating both the development and clinical translation of mRNA vaccines. This crisis-driven necessity led to the rapid deployment of mRNA vaccines, resulting in substantial reductions in COVID-19-associated morbidity and mortality, thereby establishing a historic milestone in the practical application of mRNA medicine [183]. The two mRNA vaccines that received emergency authorization were BNT162b2 and mRNA-1273 (Table 2). These vaccines use LNPs for delivery and encode a prefusion-stabilized SARS-CoV-2 spike glycoprotein while incorporating nucleoside-modified mRNA in which uridines are substituted with m1Ψ to enhance mRNA translational efficiency. Both were deployed globally and demonstrated high efficacy and acceptable safety profiles [184]. Today, this breakthrough is reflected in the expansion of mRNA vaccine research, with candidates for viruses like Nipah, rabies and Zika already being evaluated in clinical trials (Table 3). However, the development of platforms targeting bacterial and parasitic pathogens remains limited [185]. Furthermore, the continued expansion of this area is reflected in the 2024 FDA approval of an mRNA vaccine against respiratory syncytial virus (RSV) [186], representing the first mRNA vaccine approved for human use as a viral target besides SARS-CoV-2. Additionally, an influenza-specific vaccine (mRNA-1010) has recently demonstrated promising results in a phase III clinical trial (NCT05827978) [187]. mRNA vaccines are characterized by major advantages, including rapid design, high efficacy and scalability, while ongoing improvements in stability and immune durability are expected to further strengthen their clinical performance [188]. These developments position mRNA platforms as a promising technology for reshaping future strategies for infectious disease prevention and pandemic preparedness.

Beyond conventional non-replicating mRNA, saRNA represents a key technological advancement in infectious disease prevention. By incorporating viral replicase machinery, typically derived from alphaviruses, alongside the target antigen sequence, saRNA enables autonomous intracellular copy multiplication [218]. This structural feature achieves high-level, sustained antigen expression at microgram or sub-microgram levels, presenting a highly efficient mechanism for dose-sparing and minimized reactogenicity. This platform reached a key regulatory milestone following the late 2023 approval of ARCT-154 (Kostaive), marking the first clinical authorization of a saRNA-based therapeutic modality [219].

Rare genetic disorders are estimated to affect more than 300 million individuals globally; nevertheless, most of these disorders lack approved treatment options [220,221]. To address this unmet need, novel RNA-based technologies have been developed to achieve sustained clinical benefits [222]. The progression from concept to clinical application was showcased by a major milestone in 2016 with the FDA approval of an ASO therapy (nusinersen) for spinal muscular atrophy (SMA). By modulating survival of motor neuron 2 (*SMN2*) mRNA splicing, this therapy leads to remarkable clinical improvements, with long-term data supporting sustained benefits in patients [223,224]. In the past decade, a wave of approvals has resulted in 14 FDA-approved ASOs for rare diseases, including Duchenne muscular dystrophy (DMD), amyotrophic lateral sclerosis (ALS), familial chylomicronemia syndrome (FCS), and hereditary transthyretin amyloidosis (hATTR) [225]. In addition, ASO technology has facilitated the advancement of personalized medicine, enabling rapid development of tailored drugs for rare genetic disorders in several N-of-1 cases [226]. A notable example is the development of an ASO drug (milasen) for a child with Batten’s disease in 2017 [197]. Other approved ASO drugs include valeriasen, targeting a pathogenic variant in *KCNT1*, atipeksen for ataxia-telangiectasia and nL-KIF1-001 developed for the treatment of KIF1A associated neurological disorder (KAND) [227,228,229]. Another class of RNA therapeutics is siRNA-based approaches. The first such drug, approved by the FDA, was patisiran, which mediates the degradation of transthyretin (TTR) mRNA in hATTR and has demonstrated sustained clinical benefits in patients over a 5-year follow-up [83,230]. Of note, in 2025, the FDA approved two siRNA-based drugs, fitusiran for hemophilia A or B and plozasiran for FCS, the latter reducing APOC3 expression and resulting in decreased triglyceride levels [214,231]. In addition, several siRNA-based therapeutics are under clinical evaluation for the treatment of rare genetic disorders, including hereditary angioedema (NCT06960213) and ALS (NCT06556394) [232,233].

In parallel, an advanced therapeutic modality for the treatment of genetic disorders is CRISPR-dependent base editing which enables correction of pathogenic mutations at the DNA level. Initial research and clinical trials focused on CRISPR/Cas ex vivo genome editing for the therapeutic targeting of cancer, inherited blood disorders, HIV infection, type 1 diabetes and primary immunodeficiencies [234]. The first FDA-approved CRISPR/Cas9 therapy was exagamglogene autotemcel, for the treatment of inherited blood disorders (sickle cell disease and transfusion-dependent beta thalassemia) [235]. The focus of clinical research is progressively shifting from ex vivo to in vivo applications. Current clinical trials in CRISPR/Cas in vivo therapeutics are predominantly focused on monogenic hepatic disorders, leveraging the liver’s natural ability to uptake synthetic delivery platforms. A trial (NCT04601051) recently demonstrated the safety and efficacy of LNP-mediated delivery of a CRISPR/Cas9 system for the in vivo knockout of the *TTR* gene in patients with hATTR [236]. Beyond liver-related diseases, another primary target for genome editing is the eye, particularly for inherited retinal diseases (IRDs), as it displays an immune-privileged status and exhibits decreased risk of off-target effects [237]. In addition, research regarding neuromuscular disorders has been in progress, with preclinical studies successfully utilizing CRISPR to reverse pathogenic mutations of the DMD gene, associated with Duchenne muscular dystrophy [238]. However, substantial challenges related to efficiency, tissue specificity, in vivo delivery, and immunogenicity must be addressed to realize the therapeutic potential of CRISPR/Cas systems. While off-target events occur less frequently than precise editing, the risk of genotoxicity from constitutively active Cas9 necessitates precise control. Furthermore, the DNA damage response triggered by double-strand breaks can impair editing efficiency, induce cell death, or trigger innate immune responses.

Concurrently, programmable RNA editing technologies have moved into clinical evaluation as transient, safer alternatives to DNA-level double-strand interventions. By deploying engineered oligonucleotides or gRNAs to recruit endogenous cellular deaminases, such as ADARs, these systems facilitate precise, targeted single-nucleotide conversions directly on pathogenic transcripts [239]. This transcriptome-editing approach corrects point mutations or modulates protein isoforms reversibly, effectively circumventing the permanent genotoxicity and off-target challenges historically associated with traditional DNA gene editing vectors. Ongoing clinical trials are actively assessing developments to overcome the limitations and improve the overall efficiency of CRISPR/Cas therapeutics [88,240].

Cardiovascular diseases (CVDs) and metabolic disorders represent one of the leading causes of mortality worldwide, underscoring the urgent need for novel therapeutic strategies [241]. The first successful clinical application of RNA technology in CVD was the approval of inclisiran, an siRNA that reduces hepatic expression of proprotein convertase subtilisin/kexin type 9 (PCSK9), thereby lowering low-density lipoprotein cholesterol (LDL-C). Inclisiran is approved for the treatment of patients with clinical atherosclerotic cardiovascular disease (ASCVD), heterozygous familial hypercholesterolemia (FH) and primary hyperlipidemia [242,243]. Recent clinical evidence substantiated the efficacy of RNAi-based drugs, with zilebesiran targeting angiotensinogen and demonstrating significant reductions in blood pressure in the KARDIA-1 and KARDIA-2 trials, and olpasiran targeting lipoprotein(a) (Lp(a)) and achieving reductions in Lp(a) levels in patients with atherosclerotic cardiovascular disease [244,245]. In metabolic disorders, a limited number of ASOs and siRNA drugs have been approved for rare metabolic conditions [246]; however, RNA-based therapeutics are also being investigated as potential strategies for more common metabolic disorders, including obesity, with ARO-INHBE, ARO-ALK7 and WVE-007 currently under clinical evaluation [247]. Additionally, metabolic dysfunction-associated steatotic liver disease (MASLD), an emerging leading cause of liver-related morbidity and mortality, is being targeted in ongoing clinical trials (e.g., NCT05519475, NCT05648214) [248].

Cancer represents a major health burden, with projections indicating a substantial increase in cases over the next decades [249]. Despite significant advances in cancer therapies, there remains a need for more effective strategies with reduced adverse effects. mRNA-based cancer vaccines have emerged as a promising technology with numerous candidates evaluated in more than 120 clinical trials in recent years. These vaccines mainly function by facilitating intracellular expression of tumor antigens, thereby inducing an immune response against cancer cells [250]. Of note, the personalized vaccine mRNA-4157, in combination with pembrolizumab, has demonstrated encouraging clinical outcomes in patients with high-risk resected melanoma and is currently being evaluated in a phase III clinical trial (NCT05933577), as well as in non-small-cell lung cancer (NSCLC) (NCT06077760) [251]. This strategy underscores a major paradigm shift in oncology toward individualized neoantigen engineering. By analyzing a patient’s unique tumor biopsy through high-throughput sequencing and computational pipelines, private somatic mutations are identified to synthesize a tailored mRNA sequence encoding distinct, patient-specific immunogenic epitopes, thereby maximizing T-cell-mediated antitumor immunity while minimizing systemic reactogenicity. Additional promising mRNA vaccine candidates include BNT122, which is undergoing phase II clinical evaluation in colorectal cancer (NCT04486378) and pancreatic ductal adenocarcinoma (NCT05968326), and BNT113, currently being evaluated in a phase II/III trial in patients with HPV16+ recurrent or metastatic head and neck squamous cell carcinoma (HNSCC) (NCT04534205) [252,253]. Beyond mRNA vaccines, ASO- and siRNA-based therapies are emerging as novel tumor-targeted drugs, with multiple candidates currently in early phase clinical trials [254]. Notably, the only FDA-approved ASO for cancer to date is imetelstat, a first-in-class drug for adults with low- to intermediate-risk myelodysplastic syndromes (MDSs). Numerous other ASO candidates are under clinical investigation, including danvatirsen (AZD9150), which reduces the expression of STAT3, a key regulator of genes involved in cell proliferation, migration and invasion [255]. In parallel, siRNA-based approaches are also being explored as therapeutic strategies in oncology with agents such as LOcal Drug EluteR containing siRNAs against mutated (G12D) Kirsten rat sarcoma viral oncogene homolog (KRAS) in pancreatic cells (siG12D-LODER), and TKM-PLK, designed to silence polo-like kinase 1 (PLK-1) in solid tumors, both demonstrating encouraging clinical results [256].

## 5. Translational Engineering: Manufacturing, Safety and the Regulatory Landscape

The transition from laboratory-scale discovery to commercial large-scale production represents a pivotal engineering hurdle in the translation of RNA therapeutics [19]. The industrial production of RNA therapeutics is primarily divided into two methodologies: solid-phase chemical synthesis for shorter oligonucleotides, such as siRNAs and ASOs, and enzymatic IVT for long-chain transcripts including mRNA, saRNA, and circRNA [257,258]. While chemical synthesis offers high precision for molecules up to approximately 100 nucleotides, the production of longer transcripts requires the biological efficiency of bacteriophage polymerases, typically T7, T3, or SP6 [62,257]. In a standard IVT reaction, a linearized DNA template containing a specific promoter is incubated with a mixture of recombinant RNA polymerase and ribonucleoside triphosphates to produce a functional mRNA that closely mimics endogenous structures [257].

Producing long-chain RNAs presents unique engineering challenges, particularly regarding the maintenance of structural integrity and the minimization of immunogenic byproducts. For mRNA, the process must faithfully replicate five critical structural elements: the 5′ cap, the 5′ and 3′ UTRs, the CDS, and the poly(A) tail. Recent innovations have shifted from “Cap 0” structures toward “Cap 1” and “Cap 2” through co-transcriptional capping methods such as CleanCap technology [259]. This approach ensures that over 95% of transcripts are capped with an Nm, which mimics endogenous mRNA and allows the therapeutic to evade detection by PRRs [260]. For even larger constructs, such as saRNAs, which can exceed 10,000 nucleotides, the manufacturing complexity increases significantly. These molecules require the precise encoding of viral replicase proteins alongside the therapeutic gene of interest. Because long RNAs are highly susceptible to shear stress and enzymatic degradation during production, modern manufacturing utilizes optimized buffer systems and specialized bioreactors to maintain stability. Furthermore, the incorporation of modified nucleosides, such as m1Ψ, is standard practice to reduce innate immune activation and enhance the translational capacity of the transcripts [16,261].

The output of an IVT reaction is a complex mixture containing the desired RNA, residual DNA templates, proteins, and, most critically, dsRNA byproducts. dsRNA is a potent trigger for cellular sensors, such as melanoma differentiation-associated protein 5 (MDA5) and retinoic acid-inducible gene I (RIG-I), which can induce severe inflammatory responses and silence protein translation. Consequently, high-resolution purification is essential; for this reason, current industrial standards have moved beyond simple precipitation to more robust, scalable methods [262]. For instance, tangential flow filtration (TFF) is used for large-scale concentration and buffer exchange, allowing for the removal of small-molecule impurities and enzymes. Chromatography methods, including HPLC and reversed-phase HPLC (RP-HPLC), are essential for removing truncated transcripts and dsRNA. RP-HPLC at elevated temperatures is particularly effective at denaturing and separating double-stranded contaminants from single-stranded mRNA. Accordingly, enzymatic digestion using DNase I is utilized to degrade the DNA template, followed by further purification steps to ensure the final product is free of biological contaminants [257,263].

To transition these platforms from laboratory synthesis to commercial supply, strict adherence to Good Manufacturing Practice (GMP) considerations is mandatory [264]. A primary industrial challenge during scaling is maintaining absolute batch consistency, as slight variations in mixing kinetics, temperature profiles, or raw materials can lead to critical fluctuations in the polydispersity index (PDI) and encapsulation efficiency of lipid or polymeric vehicles [265]. Consequently, industrial manufacturing pipelines are increasingly migrating toward automated, closed-system bioreactors integrated with continuous-flow microfluidics to minimize operator error and secure uniform, reproducible critical quality attributes (CQAs) across manufacturing lots.

The regulatory approval of RNA drugs necessitates comprehensive analytical characterization to validate product identity, purity, safety, and stability in strict compliance with the International Council for Harmonization (ICH) guidelines [266]. Specifically, guidelines such as ICH Q6B dictate the specifications for test procedures and acceptance criteria for biotechnological products. A robust quality control (QC) matrix spans multiple orthogonal testing modalities. More precisely, the purity and integrity, cap efficiency, poly(A) tail length distribution, and the quantification of truncated transcripts or immunostimulatory dsRNA artifacts are determined using high-resolution capillary electrophoresis (CE) and RP-HPLC. Additionally, next-generation sequencing (NGS) and liquid chromatography–mass spectrometry (LC-MS) peptide mapping are utilized to confirm complete sequence fidelity and capping structure confirmation. As for the physicochemical attributes, for nanocarriers like LNPs, dynamic light scattering (DLS) and multi-angle light scattering (MALS) are mandatory to continuously track particle size distribution, zeta potential, and surface morphology. Moreover, strict limits are placed on residual plasmid DNA templates, bacteriophage polymerases, organic solvents, and endotoxins (per ICH Q3A/Q3C guidelines) to avoid contaminants and secure safety. Furthermore, in alignment with ICH Q1A (R2), rigorous real-time and accelerated stability testing schedules are vital to map degradation kinetics, establishing validated shelf-life conditions and cold-chain logistics protocols necessary for global distribution [267].

From the CMC perspective, the field is transitioning toward process analytical technology (PAT) to monitor IVT reactions in real time. This involves using sensors to track nucleoside triphosphate consumption and pH shifts, ensuring consistency across manufacturing batches [268]. As RNA therapeutics move toward N-of-1 individualized treatments, the ability to rapidly synthesize and purify long-chain transcripts in a modular, automated fashion is becoming the new benchmark for clinical translation [269].

Beyond the structural integrity of the carrier, the safety and precision of small RNA therapeutics, specifically siRNAs and ASOs, rely on mitigating hybridization-dependent off-target effects [270,271]. For siRNAs, this involves the rigorous prevention of “seed-region” matches (typically nucleotides 2–8 of the guide strand) with non-specific transcripts, a process that can mimic miRNA-like silencing and lead to significant target-independent toxicity. To suppress these effects, modern engineering strategies include the incorporation of chemical modifications, such as Nm or acyclic serinol nucleic acids (SNAs), at specific seed positions to reduce the binding affinity for off-targets while maintaining on-target potency [272]. Similarly, for ASOs, careful sequence design is required to avoid non-specific hybridization and RNase H-mediated cleavage of non-target RNAs [49].

As these therapies move through FDA and EMA regulatory pathways, manufacturers must provide comprehensive data on the biodistribution and potential toxicity of both the RNA payload and its delivery vehicle. The regulatory landscape is rapidly evolving to accommodate these “programmable” medicines, with a growing emphasis on platform-based approvals. Under this framework, the safety data for a validated delivery vehicle, such as a specific LNP or GalNAc scaffold, can be leveraged across multiple drug candidates that utilize different RNA sequences, significantly accelerating the development of N-of-1 individualized therapies. This regulatory flexibility, coupled with the advanced engineering of immunological “stealth” and high-throughput preclinical screening, is essential for the sustained growth of the RNA therapeutics sector [271].

## 6. Future Perspectives and Emerging Trends

The rapid evolution of RNA therapeutics is currently transitioning from a period of fundamental discovery to an era of computational precision and structural diversification. The integration of AI and ML is fundamentally transitioning RNA therapeutic development from a trial-and-error approach to a data-driven, predictive paradigm [273]. A primary frontier lies in AI-driven target identification and validation, where deep learning architectures process large-scale multi-omics datasets to uncover cryptic disease-associated transcripts and splicing variants that are unreachable via traditional approaches. Once a biological target is verified, AI-driven RNA sequence and codon optimization pipelines bypass traditional design spaces, which are often too vast to navigate experimentally. Generative AI models, transformer architectures, and reinforcement learning agents are now deployed to design synthetic ORFs. These computational systems intelligently substitute rare codons with synonymous preferences to match host ribosomal distributions, fine-tuning translation kinetics, while simultaneously avoiding unintended cryptic ORFs or toxic motif generation.

Concurrently, AI-guided secondary structure prediction has progressed from foundational, accuracy-limited thermodynamic folding algorithms to advanced, multi-modal neural networks. By integrating deep convolutional neural networks (CNNs), graph neural networks (GNNs), and transformer models with experimental chemical probing data (such as SHAPE), these frameworks predict complex, multidimensional RNA folding patterns and minimum free energy states with high accuracy. This structural foresight allows researchers to pre-engineer stable transcripts with enhanced intracellular longevity and lowered innate immunogenicity.

These computational tools enable the rational design of RNA sequences, allowing for the optimization of UTRs and CDS to maximize target affinity while simultaneously reducing predicted off-target binding by an estimated 40–60%. In the realm of delivery, the nearly infinite chemical space of lipid and polymer combinations is navigated through advanced ML frameworks, such as random forest, deep learning, and neural networks. These models establish quantitative structure–activity relationships (QSARs) to predict critical nanoparticle properties, including size, entrapment efficiency, and complex in vivo biodistribution patterns, with >80% accuracy [274]. By processing molecular descriptors and high-throughput screening data, AI-driven loops can compress the timeline for discovering LNPs from months to days. This computational synergy is particularly transformative for the development of individualized neoantigen cancer vaccines. AI algorithms can rapidly analyze a patient’s unique tumor phylogeny to predict epitope immunogenicity, concurrently co-optimizing the LNP chemistry to obtain a potent immune response. Furthermore, Bayesian optimization and active learning strategies are being employed to bridge the critical gap between in vitro stability and in vivo performance [275]. These methods identify hidden lipid formulations capable of crossing physiological barriers, such as the blood–brain barrier, or selectively entering specific tumor cell populations. Additionally, the broader translation of saRNA and programmable transcriptome editing, such as oligonucleotide-mediated ADAR recruitment, will expand the therapeutic window across chronic and metabolic conditions. By refining automated, microfluidics-based GMP scaling and establishing universal, platform-based regulatory approval pathways, programmable RNA is poised to cement its role as a fundamental, highly scalable pillar of precision medicine [276].

## 7. Conclusions

The evolution of RNA therapeutics redefines the parameters of modern drug development. Once hindered by inherent structural instability and severe immunogenicity, RNA has been systematically engineered and advanced into a highly programmable, versatile therapeutic platform. The unprecedented clinical success of mRNA-based vaccines during the COVID-19 pandemic has not only validated the modality, but has also established a robust translational foundation for next-generation architectures, including CRISPR-guided effectors, circRNAs, and aptamers. The future of RNA medicine will be defined by the efficient integration of AI, synthetic biology, and advanced biomaterials. Transitioning from traditional trial-and-error to data-driven, predictive design, researchers can now exploit AI to co-optimize both the RNA sequence and its nanocarrier. This synergy will drastically accelerate the development of tailored therapeutics, enabling precise transcriptomic modulation across oncology, infectious diseases and rare genetic disorders. Ultimately, RNA therapeutics provide a uniquely tunable and safer approach to disease management, establishing their role as a central pillar of precision medicine.

## Figures and Tables

**Table 1:** Overview of the advantages, limitations and clinical success examples of the RNA delivery platforms.

RNA Delivery Platform	Advantages	Challenges	Clinical Success
Lipid nanoparticles (LNPs)	Efficient endosomal escape Enhanced stability Reduced toxicity and immunogenicity Increased encapsulation efficiency Scalable manufacturing	Liver tropism PEG immunogenicity	Patisiran, the first FDA-approved siRNA drug used LNP technology. COVID-19 mRNA vaccines utilized LNPs to deliver the SARS-CoV-2 spike protein-encoding mRNA [129]
Polymer- and peptide-based nanocarriers	Structural stability Chemical flexibility for tissue-specific targeting Improved biodistribution Prolonged and controlled release of the therapeutic agent Biodegradability	Cytotoxicity Fluctuating efficiency	In preclinical/early-stage clinical status [136]
Ligand-RNA conjugates	Tissue-specific targeting Reduced nuclease degradation Reduced immunogenicity	Liver tropism	GalNAc conjugation has been utilized for the delivery of siRNA drugs givosiran, lumasiran and inclisiran [137]
Viral vectors	High transduction efficiency Long-term expression	Limited packaging capacity High immunogenicity Complex and expensive manufacturing Risk of insertional mutagenesis	Primarily utilized for gene therapy. They are also investigated for RNAi-based therapeutics in preclinical/early clinical stages [138]

**Table 2:** List of FDA-approved RNA drugs.

RNA Class	Drug	Target	Disease Indication	Year Approved
mRNAs	Tozinameran/BNT162b2	Production of SARS-CoV-2 spike protein	COVID-19	2021 [189]
	Elasomeran/ mRNA-1273	Production of SARS-CoV-2 spike protein	COVID-19	2022 [190]
	Respiratory syncytial virus (RSV) mRNA vaccine/mRNA-1345	Production of the RSV F-glycoprotein	Lower respiratory tract disease	2024 [191]
	mRNA-1283	Production of the N-terminal and receptor binding domain of the SARS-CoV-2 spike protein	COVID-19	2025 [192]
ASOs	Fomivirsen	Immediate early 2 (*IE2*) mRNA	Cytomegalovirus retinitis	1998 [193]
	Mipomersen	Apolipoprotein B (*APOB*) mRNA	Homozygous familial hypercholesterolemia	2013 [194]
	Eteplirsen	Dystrophin (*DMD*) pre-mRNA exon 51	Duchenne muscular dystrophy	2016 [195]
	Nusinersen	*SMN2* pre-mRNA	Spinal muscular atrophy	2016 [196]
	Milasen	Patient-specific mutation in major facilitator superfamily domain containing 8 (*MFSD8*)	Batten disease	2018 [197]
	Inotersen	Transthyretin (*TTR*) mRNA	Hereditary TTR amyloidosis	2018 [198]
	Golodirsen	Dystrophin (*DMD*) pre-mRNA exon 53	Duchenne muscular dystrophy	2019 [199]
	Viltolarsen	Dystrophin (*DMD*) pre-mRNA exon 53	Duchenne muscular dystrophy	2020 [200]
	Casimersen	Dystrophin (*DMD*) pre-mRNA exon 45	Duchenne muscular dystrophy	2021 [201]
	Tofersen	Superoxide dismutase 1 (*SOD1*) mRNA	Amyotrophic lateral sclerosis	2023 [202]
	Eplontersen	Transthyretin (*TTR*) mRNA	Hereditary TTR amyloidosis	2023 [203]
	Imetelstat	Telomerase RNA	Myelodysplastic syndrome	2024 [204]
	Olezarsen	Apolipoprotein C3 (*APOC3*) mRNA	Familial chylomicronemia syndrome	2024 [205]
	Donidalorsen	Kallikrein B1(*KLKB1*) mRNA	Hereditary angioedema	2025 [206]
siRNAs	Patisiran	Transthyretin (*TTR*) mRNA	Hereditary TTR amyloidosis	2018 [207]
	Givosiran	5′-aminolevulinate synthase 1 (*ALAS1*) mRNA	Acute hepatic porphyria	2019 [208]
	Lumasiran	Hydroxyacid oxidase 1 (*HAO1*) mRNA	Primary hyperoxaluria type 1	2020 [209]
	Inclisiran	Proprotein convertase subtilisin/kexin type 9 (*PCSK9*) mRNA	Hypercholesterolemia or atherosclerotic cardiovascular disease	2021 [210]
	Vutrisiran	Transthyretin (*TTR*) mRNA	Hereditary TTR amyloidosis	2022 [211]
	Nedosiran	Lactate dehydrogenase A (*LDHA*) mRNA	Primary hyperoxaluria type 1	2023 [212]
	Fitusiran	Serpin family C member 1 (*SERPINC1*) mRNA	Hemophilia A or B	2025 [213]
	Plozasiran	Apolipoprotein C3 (*APOC3*) mRNA	Familial chylomicronemia syndrome	2025 [214]
Aptamers	Pegaptanib	Vascular endothelial growth factor (VEGF)	Age-related macular degeneration	2004 [215]
	Avacincaptad pegol	Complement C5	Geographic atrophy secondary to age-related macular degeneration	2023 [216]
CRISPR/Cas	Exagamglogene autotemcel	BCL11 transcription factor A (*BCL11A*) gene	Sickle cell disease and transfusion dependent β-thalassemia	2023–2024 [217]

**Table 3:** List of RNA drugs undergoing clinical trials.

RNA Class	Drug	Target	Disease Indication	Phase	Identification Code
mRNAs	mRNA-1215	Production of the Nipah virus pre-fusion F and G glycoproteins	Nipah virus infection	I (completed)	NCT05398796
	CV7202	Production of the Rabies virus glycoprotein (RABV-G)	Rabies infection	I (completed)	NCT03713086
	mRNA-1893	Production of the RIO-U1 Zika virus premembrane and envelope (prME) structural proteins	Zikah virus infection	II (completed)	NCT04917861
	mRNA-1010	Production of hemagglutinin (HA) antigens	Seasonal influenza caused by influenza A and B viruses	III (completed)	NCT05827978
	Intismeran Autogene (mRNA-4157/V940)	Production of patient-specific neoantigens	High-risk melanoma	III	NCT05933577
			Non-small cell lung cancer (NSCLC)	III	NCT06077760
	Autogene cevumeran (BNT122/RO7198457)	Production of patient-specific neoantigens	Colorectal cancer	II	NCT04486378
			Pancreatic ductal adenocarcinoma	II	NCT05968326
	BNT113	Production of human papillomavirus type 16 (HPV16) E6 and E7 proteins	Head and neck cancer positive for HPV16 and expressing programmed cell death ligand-1 (PD-L1)	II/III	NCT04534205
ASOs	Danvatirsen (AZD9150)	Signal transducer and activator of transcription 3 (*STAT3*) mRNA	Advanced solid tumors and NSCLC	I (completed)	NCT03421353
siRNAs	ADX-324	*KLKB1* mRNA	Hereditary angioedema (Type 1 or Type 2)	III	NCT06960213
	RAG-17	*SOD1* mRNA	ALS	I	NCT06556394
	Zilebesiran (ALN-AGT01)	Hepatic angiotensinogen mRNA	Hypertension	II (completed)	NCT04936035 (KARDIA-1)
				II (completed)	NCT05103332 (KARDIA-2)
				II (completed)	NCT06272487 (KARDIA-3)
				III	NCT07181109 (ZENITH)
	Olpasiran	Apolipoprotein(a) mRNA	Atherosclerotic cardiovascular disease (ASCVD)	III	NCT05581303
	ARO-INHBE	Inhibin subunit beta E (*INHBE*) mRNA	Obesity with and without type 2 diabetes mellitus	I/IIa	NCT06700538
	ARO-ALK7	Activin A receptor type 1C (*ACVR1C*) mRNA	Obesity with and without type 2 diabetes mellitus	I/IIa	NCT06937203
	WVE-007	*INHBE* mRNA	Overweight or obesity	I/IIa	NCT06842186
	ALN-HSD	Hydroxysteroid 17-beta dehydrogenase 13 (*HSD17B13*) mRNA	Metabolic dysfunction-associated steatohepatitis (MASH)	II	NCT05519475
	ALN-PNP	Patatin like domain 3, 1-acylglycerol-3-phosphate O-acyltransferase (*PNPLA3*) mRNA	Metabolic dysfunction-associated steatotic liver disease (MASLD)	I/IIa	NCT05648214
	siG12D-LODER	Kirsten rat sarcoma viral oncogene homolog (*KRAS*) G12D mutated mRNA	Pancreatic cancer	II (unknown status)	NCT01676259
	TKM-PLK1 (TKM-080301)	Polo-like kinase 1 (*PLK-1*) mRNA	Solid tumors	I/II (completed)	NCT01262235
CRISPR/Cas	NTLA-2001	*TTR* gene	Transthyretin amyloidosis (ATTR)	I (completed)	NCT04601051

## Data Availability

No new data were created or analyzed in this study. Data sharing is not applicable to this article.

## Abbreviations

The following abbreviations are used in this manuscript:

ACVR1C	Activin A receptor type 1C
ADAR	Adenosine deaminase acting on RNA
ALS	Amyotrophic lateral sclerosis
AAV	Adeno-associated viruses
AI	Artificial intelligence
ALAS1	5′-aminolevulinate synthase 1
AOCs	Antibody-oligonucleotide conjugates
APOB	Apolipoprotein B
APOC3	Apolipoprotein C3
ASCVD	Atherosclerotic cardiovascular disease
ASGPR	Asialoglycoprotein receptor
ASOs	Antisense oligonucleotides
ATTR	Transthyretin amyloidosis
BCL11A	BCL11 transcription factor A
CAR	Chimeric antigen receptor
CDS	Coding sequence
circRNAs	Circular RNAs
CMC	Chemistry, Manufacturing and Controls
COVID-19	Coronavirus disease 2019
CPPs	Cell-penetrating peptides
CRISPR	Clustered regularly interspaced short palindromic repeats
CVDs	Cardiovascular diseases
DMD	Duchenne muscular dystrophy
dsRNA	Double-stranded RNA
EMA	European Medicines Agency
FCS	Familial chylomicronemia syndrome
FDA	Food and Drug Administration
GalNAc	N-acetylgalactosamine
gRNAs	guide RNAs
HAO1	Hydroxyacid oxidase 1
hATTR	Hereditary transthyretin amyloidosis
HIV-1	Human immunodeficiency virus type 1
HPLC	High-performance liquid chromatography
ICH	International Council for Harmonization
IE2	Immediate early 2
INHBE	Inhibin subunit beta E
IRESs	Internal ribosome entry sites
IVT	in vitro transcription
KLKB1	Kallikrein B1
KRAS	Kirsten rat sarcoma viral oncogene homolog
LDHA	Lactate dehydrogenase A
LNPs	Lipid nanoparticles
Lp(a)	Lipoprotein(a)
MASH	Metabolic dysfunction-associated steatohepatitis
MASLD	Metabolic dysfunction-associated steatotic liver disease
m1Ψ	N1-methyl-pseudouridine
m7G	N-7 methylated guanosine
MFSD8	Major facilitator superfamily domain containing 8
miRNAs	MicroRNAs
ML	Machine learning
Nm	2′-O-methylation
NSCLC	Non-small cell lung cancer
ORF	Open reading frame
PCSK9	Proprotein convertase subtilisin/kexin type 9
PD-L1	Programmed cell death ligand-1
PEG	Polyethylene glycol-modified
PLK-1	Polo-like kinase 1
PMO	Phosphorodiamidate morpholino oligomers
PRRs	Pattern recognition receptors
PS	Phosphorothioates
RBPs	RNA-binding proteins
RISC	RNA-induced silencing complex
RNAi	RNA interference
RNP	Ribonucleoprotein
RP-HPLC	Reversed-phase high-performance liquid chromatography
RSV	Respiratory syncytial virus
saRNAs	Self-amplifying RNAs
SARS-CoV-2	Severe acute respiratory syndrome coronavirus 2
SELEX	Systematic Evolution of Ligands by Exponential Enrichment
SERPINC1	Serpin family C member 1
shRNAs	Short hairpin RNAs
siRNAs	Small interfering RNAs
SMN2	Survival of motor neuron 2
SOD1	Superoxide dismutase 1
SORT	Selective organ targeting
SSOs	Splice-switching oligonucleotides
ssRNA	Single-stranded RNA
STAT3	Signal transducer and activator of transcription 3
TfR1	Transferrin receptor 1
TLRs	Toll-like receptors
TTR	Transthyretin
UTR	Untranslated region
VEGF	Vascular endothelial growth factor
Ψ	Pseudouridine
